# Report on Southern Cattle Fever1Read before the Virginia Veterinary Medical Association, Richmond, Va., January 2, 1896.

**Published:** 1896-03

**Authors:** W. H. Harbaugh

**Affiliations:** Richmond, Va.


### REPORT ON SOUTHERN CATTLE FEVER.[1]

By W. H. HARBAUGH, V.S.,
RICHMOND, VA.

DURING the past season I was unable to spare the time necessary to devote to any extended experiments in connection with this disease, but I did not entirely neglect the subject. As you will no doubt remember, I did not question the discoveries of the Bureau in regard to ticks causing the disease outside the permanently infected places, and, further, I expressed full belief in Smith's protozoal micro-organism. For the purpose of being better qualified to study the disease and its phenomena I confined my observations to the tick theory, and made as close a study of the ticks as possible under the circumstances. I was peculiarly fortunate in this respect in having the assistance of

[1] Read before the Virginia Veterinary Medical Association, Richmond, Va., January 2, 1896.

Dr. Cooper Curtice, to whose studies we are indebted for a detailed life-history of the cattle tick, the *boophilus bovis*, which is, so far as known at present, the only carrier of the cause of Texas fever.

After being able to distinguish the *boophilus bovis* from the other varieties of ticks, I made collections of them which I sent to different veterinarians outside the territory where the tick naturally exists. The arrangement with these veterinarians was to place the young ticks, hatched from the eggs of the adult ticks I sent them, on a susceptible cow and report the result, or, in other words, to repeat the experiments of the Bureau in detail.

Prof. E. P. Niles was the only veterinarian who took the trouble to carry out the experiments as agreed upon. For one reason or another the other ticks sent out were not used. The ticks I sent Prof. Niles were taken from cattle on a place where there has not been a case of the disease for at least seventeen years; but there have been no susceptible cattle added to the herd since then, the herd being kept up by breeding the cattle raised on the place. This course was found necessary by the owner. Before he commenced to breed all his own cattle he lost every animal introduced among his herd; about twenty cattle were sacrificed in his endeavors to add fresh blood to his herd.

Following is Prof. Niles's report of the experiment:

Subject: A short-horn cow about ten years old.

*October* 10*th*. Infected with a large number of young ticks.

11*th*. Infected with ticks. Temperature, 2 P.M., 101.2° F.

12*th*. Temperature, 9 A.M., 100°.

14*th*. Infected with ticks. Temperature, 2 P.M., 100.8°.

15*th*. Infected with ticks. Temperature, 3 P.M., 101.2°.

16*th*. Infected with ticks. Temperature, 3 P.M., 100.8°.

17*th*. Temperature, 5 P.M., 100.8°.

19*th*. Infected with ticks. Temperature, 5.30 P.M., 101.6°. One pair parasites in blood.

20*th*. No more ticks placed on cow. Temperature, 5.30 P.M., 101.2°.

21*st*. Temperature, 3 P.M., 101°.

23*d*. Temperature, 3 P.M., 101°. Number of parasites in blood.

25*th*. Temperature, 2 P.M., 103.4°. No record of parasites.

*26th.* Diarrhœa intense. Temperature, 9 A.M, 105.7°; 9 P.M., 107.4°. Only one pair parasites seen in blood, as mount was not properly prepared.

*27th.* Temperature, 10 A.M., 106.2°; 6 P.M., 106.6°. Few parasites seen in blood; mount not good. Profuse diarrhœa; feces colored with bile.

*28th.* Temperature, 9 A.M., 105.2°; 5 P.M., 106.4°. Number of parasites in blood; diarrhœa continues.

*29th.* Temperature, 12 M., 104.2°; 5.30 P.M., 104°. Great number of parasites in blood; bowels torpid.

*30th.* Temperature, 8.30 A.M., 101.2°; 1.30 P.M., 99.2°. Great number of parasites in blood; bowels torpid.

Cow killed on evening of 30th in dying condition. Post-mortem examination disclosed: Spleen greatly enlarged, broken down, and apparently like a mass of clotted blood when cut into. A number of petechial spots in the left ventricle of the heart. Kidneys enlarged and very dark in color. Gall bladder greatly distended with bile, as described on p. 31, Bureau of Animal Industry Report on Texas Fever. Bladder filled with very dark urine; do not know that urine was dark before death, but have every reason to suppose it was, as did not have opportunity to observe except in early stage of fever.

At the time of the experiment Prof. Niles sent me a specimen of the blood of the affected cow, which is now under the microscope for your inspection. It is a remarkably fine demonstration of Smith's micro-parasite.

Thus it will be seen that Prof. Niles's experiment confirms in every respect the work done by the Bureau. The work of the Bureau was followed in making this experiment, and, of course, nothing original is claimed for it. But it should remove from our minds any doubts as to the part played by the tick in at least being a carrier of the cause of the disease; and it may be the only carrier to places outside of the permanently infected sections, but I am still of opinion that there are other sources of infection within the permanently infected regions.

In the paper I read before this Association one year ago I called your attention to the fact that all Southern ticks were not dangerous. There are different varieties of ticks that infest animals on pasture in the South, but the species called by Dr. Curtice the *boophilus bovis* is the only one which is known to inoculate cattle with the micro-parasite of Texas fever. All other varieties are at present considered harmless. I have preserved different species for your inspection.

I must also remind you that on certain farms the *boophilus bovis* ticks do not appear to be capable of conveying the disease to susceptible cattle. On two farms in particular, a short distance below this city, on which are kept between 400 and 500 cattle, and on which susceptible cattle from the North and elsewhere are constantly being added at all seasons of the year without any regard to their susceptibility, the *boophilus bovis* species is numerous, but so far as known there never has been a case of the disease since these places were under their present ownership.

The owner attaches no importance whatever to the ticks. He does not believe they have anything to do with the disease. He has, however, his own ideas in regard to the disease and its prevention, and has perfect faith in a compound which he gives to the cattle as a preventive during the season of the year in which the disease may exist. This compound is a mixture of salt, copperas, sulphur, and nitre. It certainly does not prevent the ticks infesting his places, as there are as many ticks on his cattle as are seen on any other average herd. Other farmers have used this same compound, but their cattle became affected with the disease and died notwithstanding its use. This compound is an ancient one in this section, and is, I believe, generally known as the "old Ruffin recipe." Next season I will test its efficacy on a suitable farm.

There can be no doubt as to the genuineness of the variety of ticks which are on these places, as Dr. Curtice visited both farms and collected the ticks and sent them to Washington for experimental purposes, but I am sorry to say that he has informed me since that they failed to get a brood of young ticks from them. During the coming season I will make it my duty to collect ticks from such places to ascertain if their young will produce the disease elsewhere.

If it is established that ticks of the *boophilus bovis* species from such places cannot produce the disease, or, in other words, if it can be proven that on some places they are really harmless to susceptible cattle, such a fact will at least be a point in favor of the belief that the tick is an accidental carrier of the microparasite. The fact that the disease is transmitted to susceptible cattle with the blood of infected animals is a point in the same direction. Also, the fact that susceptible cattle may be infested with the ticks for several years in succession and do not develop the disease till the second or third year, to some extent signifies that the tick is not at all times the carrier of the disease.

If the infectious principle of the tick was of the same nature as the venom of a serpent we would not expect to find a micro-parasite as the necessary infecting principle, but as we must have the micro-parasite in order to establish the disease it is evident that it is possible for the parasite to gain access to the blood of susceptible cattle by other means than either the tick or the transmission of affected blood, which are, so far as known, the only authenticated ways the disease has been produced.

I have never heard it claimed that the tick originates the micro-parasite, but if we would accept the proposition that the tick was the only cause of the disease it would amount to almost the same thing as a claim that the tick originated the parasite. Of course it is possible, but scarcely probable, that this micro-parasite may be an organism peculiar to the tick, having to spend at least a part of its life within the tick in order to complete its round of existence. Such ideas are mere speculation, however, as next to nothing is known of the life-history of the micro-parasite.

One adult tick deposits an almost countless number of eggs from which as many young ticks are hatched, and as it is impossible for all of them to get on cattle a very large percentage must necessarily perish near where they were hatched and thereby liberate the parasites in large numbers. It is a question whether these parasites on being taken into the susceptible cow with the food or water can gain access to the blood and produce the disease. The gastric juice may guard against this source of infection, but still the question is by no means settled by the experiments of the Bureau in which they introduced the young ticks into the susceptible animals by way of the alimentary canal.

Of the greatest importance of all in connection with the study of this disease is a knowledge of the source of the protozoal parasite. At present we know absolutely nothing in regard to it. It may be said that the tick gets the parasite from an animal gradually inoculated with it by being raised on a permanently infected place, but, if so, where does the cow get it originally? On the other hand, if the cow is made immune by the tick, where does the tick get the parasite originally? It is evident that the source of the parasite is the most important feature yet to be discovered.

Recording the effect on animals placed on tick-infested farms was one of the lines of investigation I pursued during the past season. Some animals developed the disease after a few weeks' exposure, and other susceptible animals showed no signs of sickness, although well infested with ticks; while animals that had been on the places one, two, or three years, and escaped the disease in former years when cattle on the same pasture had died with it, developed the disease and died this year.

During the past season I met with cows affected with the disease on which I could discover no ticks, and upon inquiry the owners and attendants informed me that they had seen no ticks on any of their cattle. I have visited such places a month or six weeks afterward and have found ticks on all the cattle, susceptible and non-susceptible, but found no Texas fever.

Since I have had experience in hatching young ticks I will not say that a cow has no ticks on her; I can only say that after a diligent search I did not discover any; as the young ticks just hatched have the appearance of very small specks of dirt it is impossible to recognize them on a cow till they become large enough to be distinguished.

As the tick is the carrier of the cause from the places permanently infected, it must be admitted that the disease is one that may be invariably prevented in non-infected places if sufficient precautions are taken to free the cattle from ticks. Lice, mange, and scab insects are destroyed on animals, and there is no reason why ticks cannot be kept off animals.

If the disease depended solely on the presence of ticks there would be no such thing as a permanently infected place in this region. A severe winter may free a farm of all ticks.

During the early part of the fever season there was but few cases of the disease in Richmond, and it was also noticed that ticks were hard to find; but during the latter part of the season the fever prevailed to a great extent, and the ticks were still noticed to be less than in former years. Last winter was an exceedingly severe one for this section, and hence the scarcity of ticks.

During next season, among other efforts to determine the question as to whether there are other means of infection than ticks, I intend to select a farm which has been notoriously permanently infected and adopt every possible method to keep it free from ticks, and then note the effect of introducing susceptible cattle.

As to the means to be used in destroying ticks I have made no practical experiments, but it is my intention to institute experiments with the beginning of the season.

Dr. Francis, veterinarian of the Texas Agricultural Experiment Station, publishes in Bulletin No. 24, of that institution, some very practical information in regard to the methods he follows to destroy ticks on cattle. He says: "A combination of ard and sulphur or lard and kerosene gives good results." The best results obtained by him followed the use of sheep dips. He used Cannon's, Hayward's, and Little's dips, two per cent. solution (in water). On ranges where a great number of animals are to be freed from ticks he devised an unique apparatus which he describes and illustrates in Bulletin No. 24. Where the number of cattle is limited the dips are applied with sponges, mops, brushes, or syringes.

I infer from reading the Bulletin that their object in destroying ticks is not to prevent Texas fever, but in order to rid the cattle of a pest, which the tick is considered to be. When we consider the general ignorance of every particular in regard to this fatal disease manifested by those directly interested, the heavy losses sustained by cattle-owners is not at all surprising. Very few in this section realize that murrain and Texas fever mean the same disease. In view of all the information sent out by the U. S. Agricultural Department one would think that the public should be well posted on the subject, but such is not the case. Fully ninety-five per cent. of the valuable literature published by the government is wasted because it does not reach the persons who would make proper use of it, or if it does reach a certain percentage of them, it is in a shape that is incomprehensible to them, consequently is not read or studied.

There can be no doubt that if the government would issue a treatise on the subject in popular language, free from technicalities, giving the common name of the disease in each locality, and embracing the nature, characteristics, causes, so far as known, and the preventive measures, including the best means known of destroying ticks, more practical benefit would result than has followed the valuable work of the Bureau on this disease up to date. Such a publication should not be intrusted to Congressmen for distribution, but should be sent to postmasters and country merchants throughout the regions directly concerned for distribution to cattle-owners.

During the coming year Prof. Niles and myself will be your committee on this disease, and, as our work is now outlined and better understood, we hope to be able to present a report of some value to the members of the Association.

